# *Trypanosoma cruzi* produces a population of tRNA-derived small RNAs which are recruited to specific cytoplasmic granules and secreted to the extracellular medium

**Published:** 2011-01-10

**Authors:** MR Garcia Silva, M Frugier, J Tosar, A Parodi, C Rovira, C Robello, S Goldenberg, A Cayota

**Affiliations:** 1 Functional Genomics, Institut Pasteur de Montevideo, Montevideo, Uruguay; 2 Institut de Biologie Moleculaire Cellulaire., Strasbourg, France; 3 Instituto de Biologia Molecular do Parana, Fiocruz, Curitiba, Brazil; 4 Genetics, Faculty of Sciences, Universidad de la Republica, Montevideo, Uruguay; 5 Oncology, Lund University, Lund, Sweden; 6 Medicine, Faculty of Medicine, Montevideo, Montevideo, Uruguay

## 

In the last decade a new family of small regulatory RNAs (sRNAs) of 20-30 nt in length, were recognized as key players in the regulation of gene expression at the transcriptional and post-transcriptional levels. The enzymatic machinery associated with the biogenesis and effector functions of these "sRNAs" has been found in the majority of the organisms studied. The exceptions for this are some species of trypanosomatids including *Trypanosoma cruzi*. This accounts for the absence of RNA interference phenomena in these parasites. In the present work we have cloned and characterized the small RNA population from *T. cruzi,* in two of its life cycle forms: epimastigotes and metacyclic stages. Our results showed a highly represented population of small RNAs derived from de cleavage of mature tRNAs representing about 30% in epimastigotes and 40% in trypomastigotes of small RNA fraction which we dubbed mini-tRNAs. Surprisingly, more than 98% of mini-tRNAs derived from the 5' half of tRNA for Asp and Glu and localize to particular granular structures in the cytoplasm of *T.cruzi* at all stages of its life cycle. These tRNA halves seem to be related with an Argonaute protein distinctive of trypanosomatids which was recently cloned and sequenced in our laboratory. This Argonaute protein was differentially expressed through the life cycle of *T. cruzi* and its expression was accentuated by starvation. Whereas their biological significance is currently unknown this mini-tRNAs population it has been described in the last year in other organisms as *Giardia lamblia, Aspergillus fumigatus* and many mammalian cancer cells. These mini-tRNAs have also been found in the supernatant of culture of *T. cruzi* as forming part of the secretome of these organisms, which led us to speculate about a putative role of these molecules in intercellular communication. This could represent a new family of small RNAs with relevance in gene expression regulation in particular for these unicellular parasites where gene regulation is achieved principally by post-transcriptional mechanisms. The complete knowledge of gene expression regulatory pathways in this kind of parasites that concern human health could be a source of study for development of vaccines or anti-parasitic treatment in base new tools as the RNA vaccines or biotechnological drugs.

